# PTB-DDI: An Accurate and Simple Framework for Drug–Drug Interaction Prediction Based on Pre-Trained Tokenizer and BiLSTM Model

**DOI:** 10.3390/ijms252111385

**Published:** 2024-10-23

**Authors:** Jiayue Qiu, Xiao Yan, Yanan Tian, Qin Li, Xiaomeng Liu, Yuwei Yang, Henry H. Y. Tong, Huanxiang Liu

**Affiliations:** Faculty of Applied Sciences, Macao Polytechnic University, Macao SAR, China; jiayue.qiu@mpu.edu.mo (J.Q.); yan_xiao163@163.com (X.Y.); yanan.tian@mpu.edu.mo (Y.T.); qin.li@mpu.edu.mo (Q.L.); p2212241@mpu.edu.mo (X.L.); p2314424@mpu.edu.mo (Y.Y.); henrytong@mpu.edu.mo (H.H.Y.T.)

**Keywords:** deep learning, parameter-sharing, parameter-independent, DDI, website

## Abstract

The simultaneous use of two or more drugs in clinical treatment may raise the risk of a drug–drug interaction (DDI). DDI prediction is very important to avoid adverse drug events in combination therapy. Recently, deep learning methods have been applied successfully to DDI prediction and improved prediction performance. However, there are still some problems with the present models, such as low accuracy due to information loss during molecular representation or incomplete drug feature mining during the training process. Aiming at these problems, this study proposes an accurate and simple framework named PTB-DDI for drug–drug interaction prediction. The PTB-DDI framework consists of four key modules: (1) ChemBerta tokenizer for molecular representation, (2) Bidirectional Long Short-Term Memory (BiLSTM) to capture the bidirectional context-aware features of drugs, (3) Multilayer Perceptron (MLP) for mining the nonlinear relationship of drug features, and (4) interaction predictor to perform an affine transformation and final prediction. In addition, we investigate the effect of dual-mode on parameter-sharing and parameter-independent within the PTB-DDI framework. Furthermore, we conducted comprehensive experiments on the two real-world datasets (i.e., BIOSNAP and DrugBank) to evaluate PTB-DDI framework performance. The results show that our proposed framework has significant improvements over the baselines based on both datasets. Based on the BIOSNAP dataset, the AUC-ROC, PR-AUC, and F1 scores are 0.997, 0.995, and 0.984, respectively. These metrics are 0.896, 0.873, and 0.826 based on the DrugBank dataset. Then, we conduct the case studies on the three newly approved drugs by the Food and Drug Administration (FDA) in 2024 using the PTB-DDI framework in dual modes. The obtained results indicate that our proposed framework has advantages for predicting drug–drug interactions and that the dual modes of the framework complement each other. Furthermore, a free website is developed to enhance accessibility and user experience.

## 1. Introduction

In clinical medication, drug–drug interactions (DDIs) often appear when patients need to use two or more drugs simultaneously. A DDI occurs when one drug influences the pharmacokinetic or pharmacodynamic activity of another co-administered drug [1] and further leads to a change in the drug efficacy or increased risk of adverse drug events (ADEs). For example, taking aspirin and warfarin at the same time may cause serious bleeding events because the aspirin may increase the anticoagulant activities of warfarin [2]. In another typical case, when ibuprofen is combined with aspirin, the antiplatelet activities of aspirin will be decreased [3]. ADEs are a significant clinical problem in China, with nearly 1.68 million cases reported in 2020 by the National Medical Products Administration [4]. From 1999 to 2020, reported ADEs increased yearly, reaching almost 16.9 million [4]. Clinical studies suggest that about 10–30% of all ADEs are related to DDIs [2]. Thus, the identification of DDIs can decrease the risk of ADEs and is valuable to guide clinical medication. DDI prediction aims to effectively identify interactions between drugs to prevent acute ADEs during drug administration. It can help doctors and patients to achieve drug safety and effectiveness. A DDI is one of the most essential concerns in post-marketing pharmacovigilance and clinical concomitant administration [5].

The main traditional methods to identify DDI include pharmaceutical research [6], preclinical and clinical trials [7], and case reports [8]. The preclinical and clinical trials include in vitro and in vivo experiments [9]. However, these methods are costly, with incomplete consideration, and are time-consuming [10]. Deep learning methods have recently become popular in DDIs because of their convenience and capability. There are many databases that propose potential DDIs, but most of them are based not only on available clinical data but also on theoretical approaches (e.g., similarity searches) or combined information. Based on these DDI datasets, deep learning methods can directly predict the potential DDIs by learning features from various drug pairs. These methods in DDI prediction can be divided into two main categories: literature text-based and molecular structure-based.

The literature text-based methods extract drug pair relationships from known literature using text mining [11]. These methods predict potential DDI relationships based on the drug description [12]. Tari et al. [13] proposed a text-mining and automated reasoning method for predicting DDIs from Medline abstracts and others. They selected 265 drugs and extracted 17 million Medline abstracts to find implicit 5133 DDIs. Although text mining and automated inference methods can predict implicit DDIs in various texts, the predicted number of DDIs and their efficiency could be further improved. Furthermore, the automated reasoning model cannot accurately predict certain DDI cases based on the drug description alone without considering the structural properties of chemical molecules.

Compared with the literature text-based methods, the molecular structure-based methods can predict DDIs without the drug information description and promote the efficiency and accuracy of prediction [14,15,16,17]. In essence, structure-similar drugs may interact with the same target or the same metabolic enzyme. Molecular representations based on structure can be classified into the 3D spatial conformation, 2D molecular graph, and 1D Simplified Molecular-Input Line Entry System (SMILES) [18]. These structure-based representations focus on different molecular features: (1) 3D molecular conformation includes the position information of each atom in a molecule; (2) although 2D molecular graph does not contain the atomic position like the 3D conformation, it displays the topological structure information of molecules; (3) the 1D SMILES string describes the sequence information of molecules. The characterization of molecules is an important factor in determining the accuracy and reliability of molecular structure-based models.

The different ways of molecular representation have their own advantages and limitations in various works. For the 3D molecular conformation, the 3D graph and text-based neural network for the drug–drug interaction prediction (3DGT-DDI) framework [15] contains SchNet [19] for processing the molecular stereochemistry information. For the 2D molecular graph, molecular pre-training graph-based (MPG) [20] is a deep learning framework for learning molecular representation based on unlabeled molecules. For the 1D SMILES string, DeepDDI [17] predicted the unreported DDIs and described the mechanism of two drugs in the sentence by calculating the structural similarity profiles of SMILES strings. Mol2Vec [21] is inspired by Word2vec [22], which treats compound substructures as words and compounds as sentences. MolVAE [14] optimized the continuous chemical structure representation of the SMILES string in latent space based on the encoder and decoder model for DDI prediction. These previous works improve the efficiency and accuracy of DDI prediction. However, some 3D spatial conformations are unreasonable, and the generating process is time-consuming. In addition, 2D molecular graphs describe the atoms and bonds by conveying the information of neighboring atoms, resulting in information lost from distant atoms in the transmission process. The previous 1D SMILES representations are the complete molecular structure vector by directly summing up the substructure vectors, which leads to poor molecular representation and affects the predicted DDI results. Furthermore, the variational latent space process is random, which makes some molecular representations low quality. To solve the above issues, a better molecular representation way should be capable of attaining the global features and mining the 1D SMILES string implicit features in DDI prediction. In recent years, pre-trained large-scale models have gained increased attention in drug discovery [20,23,24,25]. This shows the advantages of molecular representation by pre-training millions of data to obtain the general pattern and knowledge of the molecular data. In addition, the 1D SMILES strings are easily accessible and provide a machine-readable sequence representation of the chemical structure. While the SMILES representation has its advantage for predicting potential DDIs, it may not account for interactions between biologics and small-molecule drugs.

Here, we aim to design a simple and accurate framework to predict DDIs. Firstly, our work utilizes the pre-trained large-scale model tokenizer to obtain a more comprehensive molecular representation of 1D SMILES strings. Furthermore, we consider the importance of bidirectional context-aware features for the pre-trained molecular representation. Based on these considerations, we propose an accurate and simple framework named PTB-DDI, which consists of a large pre-trained model ChemBerta [26] tokenizer, Bidirectional Long Short-Term Memory (BiLSTM) model, Multilayer Perceptron (MLP) module, and the interaction predictor. ChemBerta is an important chemistry pre-trained model trained on millions of molecules. BiLSTM [27] captures molecular bidirectional context-aware information according to its principle. Multilayer Perceptron (MLP) [28] learns the nonlinear relationship between molecules due to the capability of handling nonlinear separable problems. The interaction predictor determines whether drugs A and B have an interaction. To evaluate the performance of PTB-DDI, we conduct comprehensive and extensive experiments on the BIOSNAP and DrugBank datasets. The results of PTB-DDI show that our framework is superior to all baselines based on both datasets. Based on the BIOSNAP dataset, the AUC-ROC, PR-AUC, and F1 scores improved by 3.2%, 3.6%, and 8.7%, respectively, when compared to the MPG [20] framework. And on the DrugBank dataset, our framework shows enhancements of 4.1%, 5.3%, and 3.8% in AUC-ROC, PR-AUC, and F1 scores, respectively, compared to the CASTER [29] framework. Moreover, we investigate the effect of dual modes: (a) parameter-sharing, parameters of each module are shared in the same layer; (b) parameter-independent, each module has its parameters. In addition, we build a complimentary PTB-DDI website, supporting multiple methods for molecule input and dual modes for predicting interaction probabilities. This resource is accessible without the need for registration at https://fca_icdb.mpu.edu.mo/ptbddi/ accessed on 12 September 2024.

## 2. Results and Discussion

### 2.1. Performance Evaluation of PTB-DDI Framework and Comparison with Baselines

**Figure 1:**
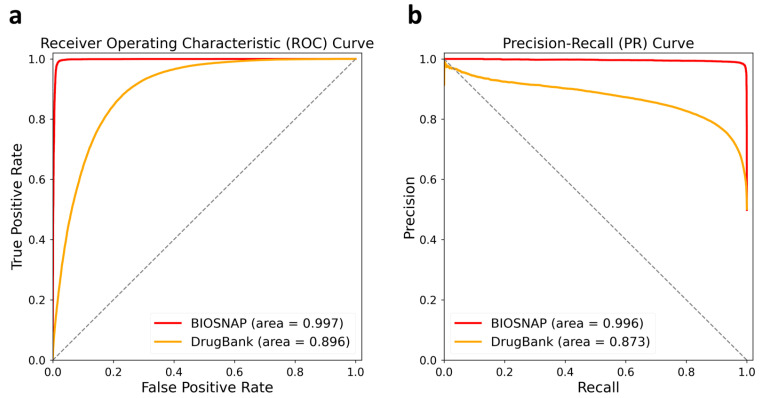
The left is (**a**) AUC-ROC curves, and the right is (**b**) PR-AUC curves of the BIOSNAP and DrugBank datasets. AUC-ROC curves are *FPR* on the *x*-axis and *TPR* on the *y*-axis, and the right shows the PR-AUC curves, in which the *x*-axis is the *R* and the *y*-axis is the *P*.

**Figure 2:**
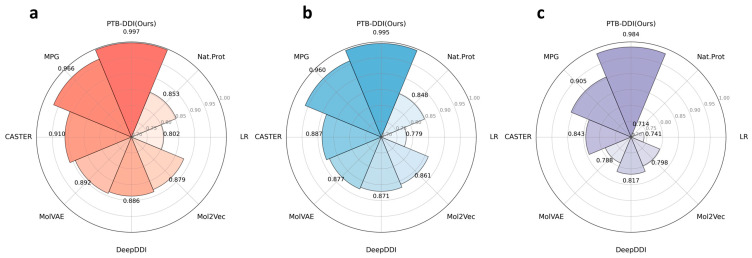
PTB-DDI test results in comparison with baselines based on the BIOSNAP dataset. (**a**) AUC-ROC, (**b**) PR-AUC, and (**c**) F1 score.

We conduct the comprehensive evaluation of our PTB-DDI framework based on the BIOSNAP [30] and DrugBank [31] datasets. To assess its effectiveness, we use the essential evaluation metrics Area Under the Receiver Operating Characteristic Curve (AUC-ROC) and Area Under the Precision-Recall Curve (PR-AUC), as Figure 1 shows. Figure 1a illustrates the AUC-ROC values, and Figure 1b delineates PR-AUC scores based on the BIOSNAP and DrugBank datasets. Furthermore, to provide a comprehensive assessment and compare our PTB-DDI framework with the state-of-the-art baselines, we evaluate the performance using three metrics: AUC-ROC, PR-AUC, and F1 score. The results of these comparisons are presented in Figure 2 and Table 1.

Figure 1a shows that the AUC-ROC score based on the BIOSNAP dataset is 0.997, which is close to 1, indicating excellent discrimination performance. The framework also performs well based on the DrugBank dataset with an AUC-ROC score of 0.896. Figure 1b shows that the PR-AUC value is 0.996 for the BIOSNAP dataset and 0.873 for the DrugBank dataset. The grey dashed line in Figure 1a,b depicts the region with a size of 0.5, representing the random prediction performance. This region is a reference for assessing the difference in the performance of the PTB-DDI framework from random prediction. The results show our PTB-DDI framework has demonstrated outstanding performance when evaluated based on the BIOSNAP dataset, showcasing its remarkable predictive capabilities. Furthermore, the framework has also exhibited commendable performance based on the DrugBank dataset, further validating its effectiveness in predicting DDIs.

To ensure reliability, we select the mean value of three independent experiment results in the framework with random seeds and fixed hyperparameters as the final performance evaluation result. As Table 1 shows, the first part displays the performance results for the BIOSNAP dataset, while the second part provides comparisons with the baseline results of the DrugBank dataset. Figure 2 is an intuitive visualization of the performance comparison between our PTB-DDI framework and other baselines based on the BIOSNAP dataset. As Figure 2 and Table 1 illustrate, the comparisons between our PTB-DDI framework and other state-of-the-art baseline models are comprehensive and detailed.

Logistic Regression (LR) [32] is the worse model than other baselines in terms of AUC-ROC and PR-AUC scores based on the BIOSNAP and DrugBank datasets. The similarity-based model [16] performs better than the LR model in the ROC-AUC and PR-AUC metrics, but the LR model performs better in the F1 score based on the two datasets. This is because the similarity-based model can capture the characteristics of drug pairs but does not learn sufficient information about each drug. Mol2Vec [21] is better than LR and similarity-based models in three metrics based on the both datasets because it learns vector representations of molecular substructures in the chemical space. Substructure vector embeddings serve as inputs into the machine learning models. MolVAE [14] improves the ROC-AUC and PR-AUC scores compared with Mol2Vec but is lower than Mol2Vec in the F1 score based on the BIOSNAP dataset, as Figure 2 clearly shows. The same performance is shown in Table 1 based on the DrugBank dataset. MolVAE can transform the molecule structures into multidimensional representations. The model encodes the SMILES string into the latent continuous representation and then passes through the decoder neural network to obtain the output. DeepDDI [17] is based on principal component analysis (PCA) and deep neural networks (DNNs) to predict DDI types. Based on the BIOSNAP dataset, DeepDDI performs better than MolVAE in the F1 score, while the AUC-ROC and PR-AUC scores are lower than the MolVAE model. Based on the DrugBank dataset, the PR-AUC score is comparable to MolVAE and the F1 score is higher than the MolVAE model, while the ROC-AUC score is lower than MolVAE. CASTER [29] outperforms previous works in three metrics based on both datasets, which is based on the encoder–decoder to extract the molecular substructure representations. MPG [20] has significantly improved the BIOSNAP dataset in three metrics compared to the other baselines, because the pre-training large-scale graph model has captured important molecular representation. This demonstrates that the pre-training large-scale model has great advantages in representing the molecules.

Table 1 demonstrates that the parameter-sharing and parameter-independent modes of the PTB-DDI framework achieve superior performance compared to all previous baselines based on the BIOSNAP and DrugBank datasets. Specifically, based on the BIOSNAP dataset, the PTB-DDI (parameter-sharing) framework exhibits notable improvements: the AUC-ROC, PR-ROC, and F1 scores are improved by 3.2%, 3.6%, and 8.7%, respectively. Similarly, the PTB-DDI (parameter-independent) demonstrates improvements of 3.1%, 3.6%, and 8.4% in these metrics. Based on the DrugBank dataset, both modes of the PTB-DDI framework also outperform previous baselines. The PTB-DDI (parameter-independent) shows the enhancements in the AUC-ROC, PR-ROC, and F1 scores by 4.1%, 5.3%, and 3.6%, respectively. Furthermore, the PTB-DDI (parameter-sharing) mode improves the F1 score by 3.8%, AUC-ROC by 3.9%, PR-ROC by 5.2%. The results with lower standard deviations highlight the consistency and reliability of our framework. In summary, these comprehensive results indicate the superiority and effectiveness of our framework in DDI prediction.

### 2.2. Discussion of Parameter-Sharing and Parameter-Independent Modes with PTB-DDI Framework

**Figure 3:**
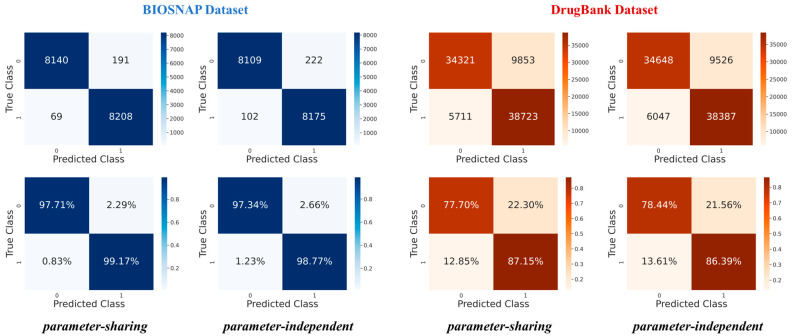
Based on the BIOSNAP (**blue**) and DrugBank (**red**) datasets, the confusion matrix (**upper**) and normalized confusion matrix (**bottom**) of the PTB-DDI framework are depicted. There are parameter-sharing and parameter-independent prediction results where the *x*-axis is the predicted value, and the *y*-axis represents the true label.

The performance of parameter-sharing and parameter-independent modes based on the BIOSNAP and DrugBank datasets are demonstrated by Table 1. Furthermore, the modes differ in the number and proportion of positive and negative predictions, which are illustrated in the confusion matrix and normalized confusion matrix of Figure 3. To ensure the robustness and accuracy of our results, we conducted three parallel experiments for both parameter-sharing and parameter-independent modes based on each dataset. The final confusion matrix represents the average of these results (Figures S1 and S2).

For the BIOSNAP dataset, the parameter-sharing mode demonstrates a higher specificity, which has fewer False Positives (FPs) of191 vs. 222 and more True Negatives (TNs) of 8140 vs. 8109 compared to the parameter-independent mode. Additionally, the parameter-sharing mode exhibits slightly lower FP and False Negative (FN) rates, at 2.29% vs. 2.66% and 0.83% vs. 1.23%, respectively, compared to the parameter-independent mode. Based on the BIOSNAP dataset, the parameter-sharing mode outperformed the parameter-independent mode, suggesting that the shared parameters may better capture the underlying patterns of this dataset. In contrast, based on the DrugBank dataset, the parameter-independent mode has a higher specificity and outperforms the parameter-sharing mode, having fewer FPs at 9526 vs. 9853 and more TNs (34,648 vs. 34,321). Moreover, the parameter-independent mode exhibits a lower FP rate (21.56% vs. 22.30%) and a higher TN rate (78.44% vs. 77.70%) than the parameter-sharing mode. Regarding FN and True Positive (TP), the parameter-sharing mode displays fewer FNs (5711 vs. 6047) and more TPs (38,723 vs. 38,387). DrugBank may contain a more complex or diverse set of drug interactions compared to BIOSNAP. The parameter-independent mode does not share parameters across different parts of the model or tasks and might be better able to process complex or diverse instances, handling variability more effectively.

The consistent observation across both datasets is that the parameter-sharing mode tends to prioritize sensitivity and minimize false negatives, which is critical in cases where failure to detect true positives can have a serious effect. The parameter-sharing mode has a smaller possibility of classifying drug pairs with interactions than non-interacting, thereby enhancing patient safety and minimizing the risk. Moreover, based on both datasets, regardless of parameter-sharing or parameter-independent, the proportion of FPs is consistently higher than the proportion of FNs, indicating that non-interacting drug pairs are more likely to be misclassified as having an interaction. The observation suggests the need for future improvement in classifying the non-interacting drug pairs. In summary, both parameter-sharing and parameter-independent modes present distinct advantages and trade-offs that should be carefully considered based on the context of application.

### 2.3. Case Study of PTB-DDI Framework Based on the Newly Approved FDA Drugs

To further evaluate the generalization ability and potential predictive power of our proposed PTB-DDI framework, we selected three new drugs approved by the Food and Drug Administration (FDA) in 2024 for case studies. The FDA newly-approved drugs are XOLREMDI™ (mavorixafor) [33], PIVYA™ (pivmecillinam) [34], and REZDIFFRA™ (resmetirom) [35], each of which holds significant implications for patients and the pharmaceutical industry. Mavorixafor is the first drug for WHIM syndrome (warts, hypogammaglobulinemia, infections, myelokathexis). Pivmecillinam, a prodrug of the beta-lactam antibiotic mecillinam, is indicated for the treatment of uncomplicated urinary tract infections (UTIs). Resmetirom, which treats patients with noncirrhotic nonalcoholic steatohepatitis (NASH) having moderate-to-advanced liver fibrosis, is the first FDA-approved therapy for this disease. The three drugs, which were not included in previous datasets, are suitable for evaluating the potential predictive power of our framework. However, processed DDI pairs and non-interacting pairs are unavailable in accessible resources, and only extensive package inserts are provided. We thoroughly reviewed the complete package inserts for these newly approved drugs and curated them to identify DDIs and non-interacting pairs.

**Figure 4:**
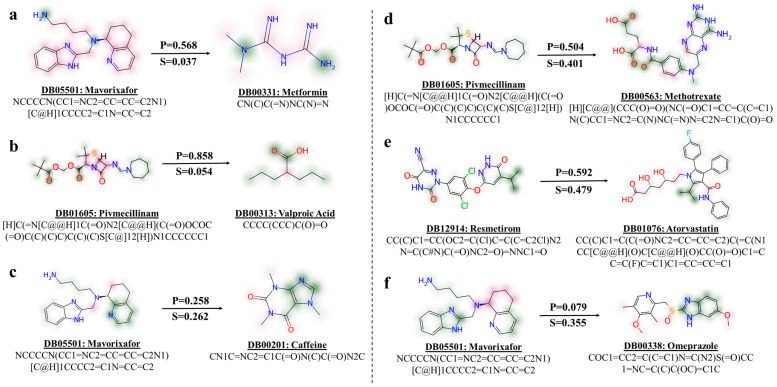
The case studies include the prediction of drug pairs (**a**–**c**) using the parameter-sharing mode of the PTB-DDI framework, as well as the prediction of drug pairs (**d**–**f**) using the parameter-independent mode. In the visual representation, the green atom indicates a positive contribution to the similarity; when the bits are removed, the similarity decreases. In contrast, the pink atom denotes a negative contribution to the similarity. The intensity of the color indicates the strength of the similarity contribution, with darker hues indicating a greater contribution and lighter hues representing a smaller contribution.

In the context of real-world applications, accurately identifying DDIs is of paramount importance compared to detecting non-interacting drug pairs. From the package inserts of newly approved drugs, we select four DDIs from the newly approved drug package inserts and two non-interacting drug pairs for analysis. As illustrated in Figure 4, subfigures (a), (b), (d), and (e) represent DDIs labeled as ‘1’, and subfigures (c) and (f) represent non-interacting pairs labeled as ‘0’. We annotated each drug with its corresponding drug names, DrugBank ID, and SMILES string to facilitate clear identification.

The case studies include the following:

- Mavorixafor and metformin (DB05501 and DB00331, respectively): mavorixafor is reported to reduce the effectiveness of metformin by decreasing its maximum serum concentration (Cmax) and area under the curve (AUC) [33].
- Pivmecillinam and valproic acid (DB01605 and DB00313, respectively): concurrent use is discouraged due to the risk of carnitine depletion, and monitoring for adverse drug events (ADEs) is recommended if co-administration is necessary [34].
- Mavorixafor and caffeine (DB05501 and DB00201, respectively): the pharmacokinetics of caffeine show no clinically significant differences when administered with mavorixafor [33].
- Pivmecillinam and methotrexate (DB01605 and DB00563, respectively): pivmecillinam is known to reduce the clearance of methotrexate from the body [34].
- Resmetirom and atorvastatin (DB12914 and DB01076, respectively): resmetirom increases the exposure to atorvastatin when used concurrently [35].
- Mavorixafor and omeprazole (DB05501 and DB00338, respectively): No clinically significant changes are noted in the pharmacokinetics of omeprazole when co-administered with mavorixafor [33].

We utilize the specified case studies to evaluate the effectiveness of our dual-mode framework. The parameter-sharing mode is assessed based on the cases (a), (b), and (c), and the parameter-independent mode uses drug pairs (c), (d), and (e), respectively. The PTB-DDI (parameter-sharing) framework calculates the interaction probabilities as follows: mavorixafor and metformin yield a probability *P* of 0.568, and pivmecillinam and valproic acid have a probability of 0.858, as depicted in Figure 4a,b. These probabilities exceed the threshold of 0.5, suggesting the presence of interactions and confirming the accuracy of the predictions. Moreover, within the same parameter-sharing mode, the interaction probability between mavorixafor and caffeine is 0.258 as Figure 4c shows, which is below 0.5, indicating their non-interacting type as accurately predicted. Furthermore, the parameter-independent mode accurately predicts the interaction between pivmecillinam and methotrexate with an interaction probability of 0.504, as illustrated in Figure 4d. Similarly, the interaction probability for resmetirom and atorvastatin is calculated at 0.592, as shown in Figure 4e, indicating an interaction. Additionally, mavorixafor and omeprazole exhibit an interaction probability of 0.079, further affirming the efficacy of the framework in correctly identifying the non-interacting pair.

To further compare the drug molecules, we use the Tanimoto coefficient [36] to measure the similarity and plot the similarity map [37] to visualize atomic contributions between the drug fingerprints. The findings surpassed our initial expectations. Initially, we hypothesized that the parameter-sharing mode would correctly predict cases where drug pairs exhibited higher similarity, while the parameter-independent mode would accurately predict cases of lower similarity. Contrary to our expectations, the parameter-sharing mode accurately predicts cases with very low similarity, with molecular fingerprint similarities approaching zero; for example, the Tanimoto similarity *S* between mavorixafor and metformin is 0.037, and that between pivmecillinam and valproic acid is 0.054. On the other hand, the parameter-independent mode effectively predicts cases with higher similarity, for example, the Tanimoto similarity S of 0.401 between pivmecillinam and methotrexate and 0.479 between resmetirom and atorvastatin. These observations suggest that drug–drug interactions are governed by more complex mechanisms rather than merely the similarity of their molecular fingerprints. Moreover, parameter-sharing allows the framework to learn identical weights for different parts of the sequences, enabling continuous mutual learning between the molecular sequences. This capability allows the framework to better capture local patterns within the data, enhancing its expressive power and generalizability, rather than solely relying on similarity to determine interactions. Conversely, in the parameter-independent mode, features of each drug within the pairs are learned separately. When these features exhibit high similarity, they may be considered in the criteria for assessing potential interactions.

Currently, predicting DDIs and non-interactions for newly approved drugs presents a significant challenge for AI models. This challenge stems from the limited generalization ability of existing models, which do not yet match the leading industry specialists, coupled with the variable quality of DDI datasets. Our framework has demonstrated the capability to accurately predict DDIs for some new drugs, and we aim to provide support to pharmaceutical companies and academic research institutions in the future. We recommend that these institutions adopt the dual mode of our framework to enhance the screening of DDIs. Each of the two complementary modes of our framework has unique advantages. By integrating their predictions, it is possible to identify DDI pairs that might be overlooked by a single mode, thus enhancing the accuracy and completeness of DDI predictions.

### 2.4. PTB-DDI Website for Predicting Drug–Drug Interactions

To facilitate easy access to DDI predictions and to promote our PTB-DDI framework, we developed a user-friendly DDI prediction website. In contrast to well-known DDI websites, such as Lexi-Interact^®^ [38], Micromedex Drug Interactions^®^ [38], and iFacts^®^ [38], which are commercial platforms, or Medscape [38], Epocrates [38], and DDInter [39] that provide free access, our website uniquely aims at the identification of potential DDIs. Compared to these existing websites that offer extensive, authoritative curated data for known drugs and are primarily aimed at clinical decision support, our website specifically fills a critical gap by concentrating on the exploration of potential DDIs, thereby expand the scope of existing DDI resources by providing insights into potential DDIs. Our PTB-DDI website model, which has been trained on 283,549 DDI data, is capable of accurately predicting some unknown DDIs of new drugs according to the case study results. The website suits professionals who have the fundamental knowledge of reference and pharmaceutical companies or academic research institutions to reduce their costs. It can be a supplementary and double-check method for DDI experiments to keep accuracy; meanwhile, as a tool to quickly screen the huge number possible DDI pairs, after screening out the possible DDIs, reverifying them based on the experiment, it can save much time and cost. The PTB-DDI website is the first free open-source DDI website for predicting the interaction probability of unknown DDIs, without registration, accessible at https://fca_icdb.mpu.edu.mo/ptbddi/, accessed on 12 September 2024.

The PTB-DDI website is developed using the Flask Python web framework, enhanced with Bootstrap and WTForms for robustness and user interaction. The web interface is designed using HTML5, CSS3, and JavaScript, ensuring a dynamic user experience. Molecular validity verification is conducted using the RDKit [40] library 2024.3.3, and data-manipulation tasks are facilitated using the Pandas package 1.3.5. The JSME.js [41] library provides functionalities for drawing and editing molecular structures directly within the browser. Comprehensive testing across multiple operating systems and web browsers has ensured that the functionality of the website is applicable across all platforms.

**Figure 5:**
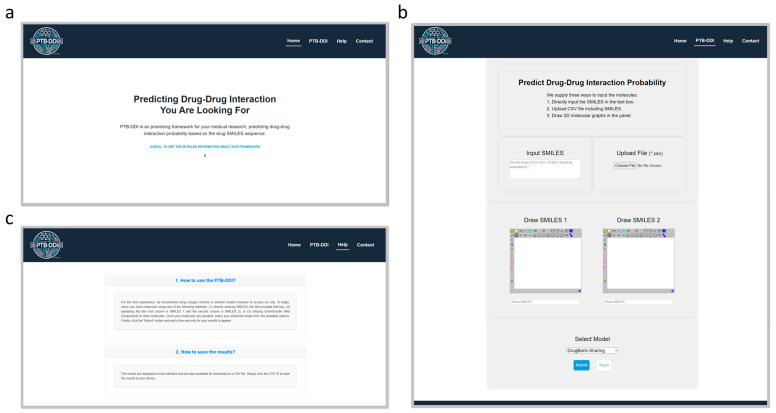
The web interface of PTB-DDI. (**a**) Introduction page about the website goal. (**b**) Main functional page of the website. There are three ways to input molecules for predicting DDIs: (1) directly input the SMILES into the text box, (2) upload a CSV file including SMILES, and (3) draw 2D molecular graphs in the panel. (**c**) Help page for users.

As illustrated in Figure 5a, the primary objective of the site is to predict DDI interaction probabilities. However, due to spatial constraints within this document, further introductory material is available directly on the site. The principle operational page, depicted in Figure 5b, offers three input methods for molecules: direct input, CSV file upload, and an interactive molecular drawer. The input box facilitates the processing of known SMILES string drug pairs directly. The CSV upload method is configured to accept files with two columns representing the SMILES strings of the first and second drugs, respectively. For optimal performance, we recommend that files should not exceed 5 MB to ensure a rapid response and feedback. Furthermore, the molecular drawer method permits users to edit and revise 2D molecular structures, which can be automatically converted into SMILES strings. Users can select between two computational modes within our dual-mode framework: ‘DrugBank-Sharing’ or ‘DrugBank-Independent’. These modes correspond to the parameter-sharing and parameter-independent models, respectively, trained using the DrugBank dataset. Results will be displayed after data submission, indicating the interaction probabilities. In addition, a ‘Download’ option allows the user to export their results in CSV format. Detailed guidance on utilizing the website is available on the ‘Help’ page, as shown in Figure 5c. This PTB-DDI website enhances the predictive capabilities for DDIs and supports the broader scientific community in advancing the management of potential pharmacological interactions.

Nevertheless, the PTB-DDI website still has several limitations that necessitate future iterative improvements. Firstly, this website cannot guarantee accurate prediction for all data; thus, all results should be considered as reference material for professional institutions and specialists rather than definitive results. Secondly, while the website provides interaction probabilities for drug pairs, it does not furnish explanations for the mechanisms underlying the DDIs. Thirdly, our current framework ignores the drug dose when predicting DDIs. If this framework is to be applied clinically in the future, incorporating dose information is vital for drug metabolism and pharmacokinetic studies. Moving forward, we plan to develop a more comprehensive and generalized model for DDI prediction, regularly update the website, and incorporate additional functionalities to elucidate the reasons behind the DDI predictions.

## 3. Materials and Methods

### 3.1. Materials and Datasets

Two DDI datasets (i.e., BIOSNAP [30] and DrugBank [31]) processed by Huang et al. [29] were used for the development and evaluation of the DDI prediction framework. These datasets contain drug SMILES strings and their interaction labels. The training and validation set accounts for 80% of the total dataset. The test dataset is 20%. The ratio between the training and validation sets is 8:2. Consistent with all baseline comparisons, the BIOSNAP and DrugBank datasets are split in the same way. Table 2 shows the details of the BIOSNAP and DrugBank datasets.

The BIOSANP dataset contains 83,040 DDI pairs, of which 41,520 are positive labels and 41,520 are negative labels. The positive labels indicate that one drug pair could alter the activity, absorption, distribution, metabolism, excretion, toxicity, and other characteristics of another drug, while the negative labels mean the drug pair will not affect each other. The DrugBank dataset has 443,046 DDIs, which is larger than the BIONSNAP dataset. It has 221,253 positive labels and 221,253 negative labels. The number of positive and negative labels is equal, forming a balanced dataset. It is beneficial to avoid the bias of the different label types for evaluating the framework performance.

### 3.2. PTB-DDI Framework Overall Architecture

**Figure 6:**
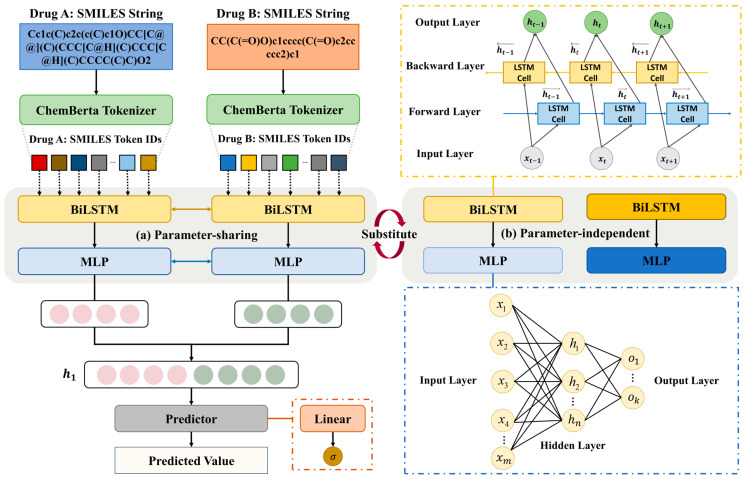
The framework architecture of the PTB-DDI consists of the ChemBerta tokenizer, BiLSTM module, and MLP module. It has dual modes: (**a**) parameter-sharing (the default mode), the identical color rectangles mean the parameters of modules are the same; (**b**) parameter-independent, the different color rectangles denote that the modules have their own parameters. The parameter-independent and the parameter-sharing can be interchanged.

As Figure 6 shows, the PTB-DDI framework mainly contains four modules: ChemBerta tokenizer, BiLSTM, MLP, and predictor. The 1D SMILES pair crosses different modules in the arrow direction. The BiLSTM module consists of an input layer, forward layer, backward layer, and output layer. Each token ID generated by the ChemBerta tokenizer is inputted into the BiLSTM to obtain the forward hidden state and backward hidden state. Then, it concatenates the forward hidden state and backward hidden state to obtain the hidden state. The hidden state is processed by the MLP module. Then, it concatenates the two drug representations to obtain the final hidden state $h_{1}$. Finally, $h_{1}$ is injected into the predictor for DDI prediction. The predictor is composed of the linear layer and sigmoid activation function [42]. The linear layer performs an affine transformation of data and the sigmoid activation function transforms the data into [0, 1] to obtain the final DDI prediction.

The overall framework is simple, but it is accurate and saves computational resources. Furthermore, PTB-DDI is designed with dual modes: (a) parameter-sharing and (b) parameter-independent, as shown in Figure 6. No study to date has investigated the effects of parameter-sharing and parameter-independent on DDI prediction tasks based on the drug structure. Therefore, we are interested in exploring the effect of parameter-sharing and parameter-independent on the PTB-DDI framework. The neural network layers share parameters that not only improve the efficiency [43] but also capture the common features of drugs A and B. The independent parameters of the layers also have advantages. When processing the structure of drugs A and B, layers can adapt to the distinct features between them.

#### 3.2.1. ChemBerta Tokenizer for Molecular Representation

ChemBerta [26] is a self-supervised pre-trained large-scale model based on RoBERTa [44] for chemical SMILES strings. The model learns the common molecular characteristics from the labeled and unlabeled millions of molecules. The weights of ChemBerta are pre-trained on the various datasets, including ZINC [45] and PubChem [46], to learn the molecular representation. Then, fine-tune the ChemBerta model on the small molecular property datasets (e.g., toxicity, solubility, drug-likeness, and synthesis accessibility). ChemBerta is used in many downstream tasks, such as predicting the masked atoms, functional groups, and molecular properties.

Due to the previous successful application [23], we attempt to adopt ChemBerta for the DDI prediction task through effective transfer learning [47]. Although ChemBerta has enormous power in many chemistry tasks, the complexity and resource consumption of the whole model are stumbling blocks. The model contains 12 attention heads and six layers, between 5 M and 46 M parameters [48]. To reduce computational resource consumption, we only use the ChemBerta tokenizer for drug representation. The ChemBerta tokenizer adopted the Byte-Pair Encoder (BPE) [49] algorithm to represent the molecules. It processes the raw SMILES strings into specific token IDs. BPE creates the representation of atoms and functional groups in the molecule. Rare and unfamiliar functional groups are split into known atoms. The BPE algorithm determines the optimal segmentation of the functional groups by iteratively merging the frequent atoms. As shown in Figure 6, the ChemBerta tokenizer parses the SMILES strings of drugs A and B into the different token IDs, respectively. As the molecular representation, token IDs are inputted into the BiLSTM model.

#### 3.2.2. BiLSTM to Learn Molecular Bidirectional Contextual Features

Bidirectional Long Short-Term Memory (BiLSTM) is a bidirectional structure that contains the forward Long Short-Term Memory (LSTM) [50] layer and backward LSTM layer. It allows the model to propagate forward and backward simultaneously during training. The flow direction of input data into the forward LSTM layer and backward LSTM layer is shown in Figure 6. For example, the token id $x_{t}$ as the input is processed by the LSTM cells. The forward layer processes$\;x_{t}$ in the LSTM cell to obtain the forward hidden state $\vec{h_{t}}$. Meanwhile, the$\;x_{t}$ passes through the backward layer to obtain the backward hidden state $\overset{\leftarrow }{h_{t}}$. Finally, the forward and backward hidden states $\vec{h_{t}}$ and $\overset{\leftarrow }{h_{t}}$ will be concatenated to output the final hidden state $h_{t}$ as Equation (1) shows:

(1) $$h_{t}=Concatenate\left(\vec{h_{t}}\;,\;\overset{\leftarrow }{h_{t}}\right)$$

**Figure 7:**
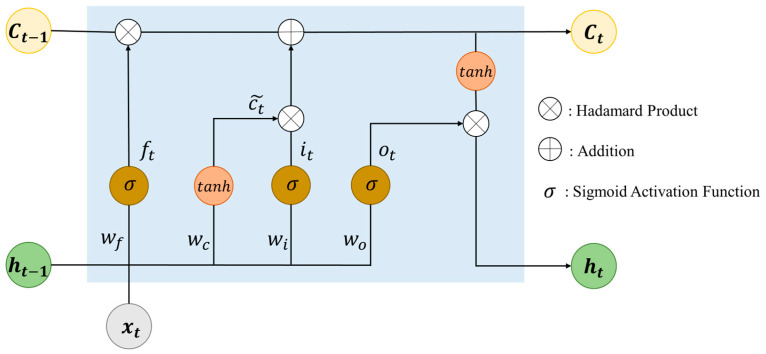
LSTM cell calculation procedure. It obtains the memory cell state $\left(c_{t-1},c_{t}\right)$ and hidden state $\left(h_{t-1},h_{t}\right)$.

LSTM overcomes the recurrent neural network (RNN) model that forgets starting input information during training [51]. As shown in Figure 7, it can store long-term data information because of the long-range LSTM cell. It contains the input gate, forget gate, and output gate. The input gate $i_{t}$ determines whether a new token is encoded to overwrite the old token. The forget gate $f_{t}$ controls whether useless information is discarded. If there is a state of an opened forget gate and closed input gate, the previous data information is stored for as long as possible. The state of the output gate $o_{t}$ does not affect other gates. It individually determines the open state or closed state by itself. The following equations describe the calculation steps of gates:

(2) $$f_{t}=\sigma (w_{f}\cdot \left[x_{t},\;{\;h}_{t-1}\right]+b_{f})$$

where $w_{f}$ is the weight and $b_{f}$ is the bias in the forget gate. The symbol σ stands for the sigmoid activation function, and $h_{t-1}$ represents the hidden state at the $\left(t-1\right)$ step.

The input gate, output gate, and cell input gate $\tilde{c_{t}}$ are calculated by the following:

(3) $$i_{t}=\sigma (w_{i}\left[x_{t},\;{\;h}_{t-1}\right]+b_{i})$$

(4) $$o_{t}=\sigma (w_{o}\left[x_{t},\;{\;h}_{t-1}\right]+b_{o})$$

(5) $$\tilde{c_{t}}=tanh(w_{c}\left[x_{t},\;{\;h}_{t-1}\right]+b_{c}\;)$$

where $b_{i}$, $b_{o}$, and $b_{c}$ denote the bias and $w_{i}$, $w_{o}$, and $w_{c}$ stand for the weights in the input gate, output gate, and cell input gate, respectively.

Finally, the cell output gate $c_{t}$ and next step hidden state $h_{t}$ are given by the following:

(6) $$c_{t}=f_{t}\;⊙{\;c}_{t-1}+i_{t}\;⊙\;\tilde{c_{t}}$$

(7) $$h_{t}=o_{t}\;⊙\tanh\left(c_{t}\right)$$

where the operator ⊙ represents the Hadamard product.

In summary, BiLSTM sequentially encodes the input token IDs to learn long-term dependency. It is particularly effective in learning bidirectional contextual features of the drug representation.

#### 3.2.3. MLP for Mining the Nonlinear Relationship of Drug Features

Multilayer Perceptron (MLP) combines one or more hidden layers based on a single neural network to learn nonlinear relationships between inputs and outputs. As shown in Figure 6, MLP contains three layers: the input layer, hidden layer, and output layer. There are m units in the input layer, n hidden units in the hidden layer, and k units in the output layer. The input layer does not perform any calculations in the training process. The input and hidden layers are fully connected as the same as the hidden and output layers. However, the fully connected layer only affinely transforms data, so we use the nonlinear function (activation function) to change the hidden state. The activation function between the fully connected layers is the Rectified Linear Unit (ReLU) [52]. The hidden state H and output O are calculated as follows:

(8) $$H=ReLU(W_{h}x+b_{h})$$

(9) $$O=HW_{o}+b_{o}$$

where x is the input data, $W_{h}$ represents the weight of the hidden layer, and $W_{o}$ is the weight of output layer. The symbol $b_{h}$ stands for the bias of the hidden layer, and $b_{o}$ is the bias of the output layer in MLP.

### 3.3. Effects of Parameter-Sharing and Parameter-Independent

Parameter-sharing and parameter-independent are two different modes of processing inputs in our framework. Parameter-sharing replicates sets of weights and bias values in layers or models [53]. It allows the child models or layers to transform empirical knowledge from previous data to the next one and save computational resources [53]. This method improves the model performance when used in multitask learning [21,32]. On the other hand, parameter-independent updates parameters individually within layers or models. It enhances the flexibility of the model and facilitates layers to mine distinct input information.

In medicinal chemistry, if drugs A and B have high similarity values, they are more likely to have a drug–drug interaction as they are more likely to interact with the same target [54]. Besides, if drug A interacts with B, and drug C is similar to B, then drug A has a high probability of interacting with C [55]. As shown in Figure 6a, we share parameters in the BiLSM and MLP modules to discover the commonalities between drugs. The parameter-sharing mode allows drugs A and B to interact and influence each other within the layer, which sequentially updates the weights and biases. Furthermore, as Figure 6b shows, BiLSTM and MLP modules have their own parameters. Independent parameters enable the modules to adjust the parameters of drugs A and B in the training process for learning their features, respectively. Parameter-sharing and parameter-independent have different advantages. In this study, we will investigate the performance and effect of parameter-sharing and parameter-independent in our PTB-DDI framework.

### 3.4. Experimental Setting of PTB-DDI Framework

The programming language is Python, and the virtual running environment is built on the Conda. Python version is 3.10.0 and Pytorch version is 1.13.1 with CUDA 11.6 version. The packages include Transformers 4.24.0, Matplotlib 3.5.1, Seaborn 0.12.2, and Numpy 1.22.0. The obtained optimal hyperparameter combination setting is given in Table S1.

Table S1 shows the obtained optimal hyperparameter combination setting of the PTB-DDI framework. The max sequence length of token IDs generated by the ChemBerta tokenizer is 512. The AdamW [53] optimizer has a learning rate of 2 × 10^−5^ and weight decay of 2 × 10^−4^ based on the BIOSNAP dataset, while it has a learning rate of 2 × 10^−5^ and weight decay of 1 × 10^−2^ based on DrugBank. The batch size is 8 for the BIOSNAP and 16 for the larger DrugBank dataset. The step learning rate (StepLR) scheduler has a gamma of 0.8 and a step size of 10. The total number of training epochs is 30. In the BiLSTM model, the input layer has 512 units, and the hidden layer dimension is 256. In the MLP module, the input layer has 256 neurons, the hidden units are 128, and the output layer has four units. In the predictor, the input dimension of the linear layer is eight and the output dimension is 1.

### 3.5. Performance Metrics

The performance of our proposed framework PTB-DDI is evaluated based on three metrics: F1 score, AUC-ROC, and PR-AUC.

The F1 score evaluates the performance of each class in the dataset. It is calculated based on the precision (P) and recall (R) values. The P-value measures how many drug–drug interactions are correct in all predicted positive labels. The R-value quantifies the number of the framework that is accurately predicted in all true drug–drug interactions (label 1). These metrics are calculated by the following:

(10) $$P=\frac{TP}{TP+FP}$$

(11) $$R=\frac{TP}{TP+FN}$$

(12) $$F1=\frac{2PR}{P+R}$$

where TP represents the true positive, FP means the false positive, and FN is the false negative.

The AUC-ROC is a metric used to evaluate the overall performance of the framework. The AUC-ROC curve represents the trade-off between the true positive rate (TPR) and the false positive rate (FPR). The higher TPR and lower FPR mean that the upper left curve has more area. It indicates that the AUC-ROC value is high and has better discrimination performance for the framework. An AUC-ROC value closer to 1 shows excellent performance. The *TPR* (sensitivity) is the same as recall. The *FPR* (1—specificity) is calculated as follows:

(13) $$FPR=\frac{FP}{FP+TN}$$

where TN is the true negative.

The PR-AUC is another performance metric to evaluate the framework. It plots the P-value against the R-value to depict the trade-off between precision and recall. In addition, it is a measure of correctly identifying positive instances while minimizing false positives. The higher PR-AUC value indicates better model performance, if the value near 1 indicates a perfect trade-off between precision and recall.

Moreover, we selected the Binary Cross Entropy Loss (BCELoss) criterion to avoid the model overfitting. The BCELoss measures the binary cross entropy between input and predicted labels in the training and validation processes. The calculation formula is as follows:

(14) $${Loss}_{n}=-w_{n}\;[t_{n}\cdot \log p_{n}+\left(1-t_{n}\right)\;\cdot \log\left(1-p_{n}\right)]$$

where $w_{n}$ is the rescaling weight for elements, $t_{n}$ is the true label, and $p_{n}$ is the predicted label.

## 4. Conclusions

This paper proposes an accurate and simple PTB-DDI framework for drug–drug interaction prediction. This framework consists of the ChemBerta tokenizer, BiLSTM model, MLP module, and predictor. It processes molecules using 1D SMILES strings using the pre-trained large-scale model tokenizer. In addition, it considers the importance of bidirectional context-aware features on the pre-trained molecular representation. The PTB-DDI framework performance is superior to all baseline models based on the BIOSNAP and DrugBank datasets. Specifically, it enhances the AUC-ROC, PR-AUC, and F1 scores on the BIOSNAP dataset by 3.2%, 3.6%, and 8.7%, respectively, Similarly, based on the DrugBank dataset, it shows improvements of 4.1% in the AUC-ROC, 5.3% in the PR-AUC, and 3.8% in the F1 score. This paper also explores the effects of employing parameter-sharing and parameter-independent modes within the framework. The dual modes of the PTB-DDI framework have their advantages. The parameter-sharing mode saves computational resources and achieves comparable results to the parameter-independent mode. On the other hand, the parameter-independent framework is more flexible in tuning the different parameters for drug pairs and occasionally surpasses the parameter-sharing mode in performance. To facilitate predicting DDIs and enhance accessibility, we develop a user-friendly website at https://fca_icdb.mpu.edu.mo/ptbddi/, accessed on 12 September 2024, supporting the practical application of the PTB-DDI framework.

## Figures and Tables

**Table 1:** The comparison of prediction performance between the PTB-DDI framework and the state-of-the-art baseline models.

Model	AUC-ROC	PR-AUC	F1
BIOSNAP Dataset			
LR [32]	0.802 (±0.001)	0.779 (±0.001)	0.741 (±0.002)
Nat.Prot [16]	0.853 (±0.001)	0.848 (±0.001)	0.714 (±0.001)
Mol2Vec [21]	0.879 (±0.006)	0.861 (±0.005)	0.798 (±0.007)
MolVAE [14]	0.892 (±0.009)	0.877 (±0.009)	0.788 (±0.033)
DeepDDI [17]	0.886 (±0.007)	0.871 (±0.007)	0.817 (±0.007)
CASTER [29]	0.910 (±0.005)	0.887 (±0.008)	0.843 (±0.005)
MPG [20]	0.966 (±0.004)	0.960 (±0.004)	0.905 (±0.008)
PTB-DDI (parameter-independent)	0.996 (±0.0002)	0.995 (±0.0004)	0.981 (±0.0003)
PTB-DDI (parameter-sharing)	0.997 (±0.0001)	0.995 (±0.0003)	0.984 (±0.0009)
**DrugBank Dataset**			
LR [32]	0.774 (±0.003)	0.745 (±0.005)	0.719 (±0.006)
Nat.Prot [16]	0.786 (±0.003)	0.753 (±0.003)	0.709 (±0.004)
Mol2Vec [21]	0.849 (±0.004)	0.828 (±0.006)	0.775 (±0.004)
MolVAE [14]	0.852 (±0.006)	0.828 (±0.009)	0.769 (±0.031)
DeepDDI [17]	0.844 (±0.003)	0.828 (±0.002)	0.772 (±0.006)
CASTER [29]	0.861 (±0.005)	0.829 (±0.003)	0.796 (±0.007)
PTB-DDI (parameter-sharing)	0.895 (±0.0005)	0.872 (±0.0004)	0.826 (±0.0005)
PTB-DDI (parameter-independent)	0.896 (±0.00009)	0.873 (±0.0004)	0.825 (±0.0004)

**Table 2:** The details of BIOSNAP and DrugBank datasets.

Dataset	#Total	#Train	#Valid	#Test	#Positive	#Negative
BIOSNAP	83,040	53,152	13,280	16,608	41,520	41,520
DrugBank	443,046	283,549	70,887	88,610	221,523	221,523

## Data Availability

The code and data are online at the GitHub repository: https://github.com/drunkprogrammer/PTB-DDI, accessed on 6 June 2024.

## Supplementary Materials

The following supporting information can be downloaded at: https://www.mdpi.com/article/10.3390/ijms252111385/s1.

## References

[1] Miranda V., Fede A., Nobuo M., Ayres V., Giglio A., Miranda M., Riechelmann R.P. (2011). Adverse Drug Reactions and Drug Interactions as Causes of Hospital Admission in Oncology. J. Pain Symptom Manag..

[2] Dechanont S., Maphanta S., Butthum B., Kongkaew C. (2014). Hospital Admissions/Visits Associated with Drug–Drug Interactions: A Systematic Review and Meta-analysis. Pharmacoepidemiol. Drug.

[3] So C.H., Eckman M.H. (2017). Combined Aspirin and Anticoagulant Therapy in Patients with Atrial Fibrillation. J. Thromb Thrombolysis.

[4] Shibata K., Akagi Y., Nozawa N., Shimomura H., Aoyama T. (2017). Influence of Nonsteroidal Anti-Inflammatory Drugs on Aspirin’s Antiplatelet Effects and Suggestion of the Most Suitable Time for Administration of Both Agents without Resulting in Interaction. J. Pharm. Health Care. Sci..

[5] National Medical Products Administration (2021). National Adverse Drug Event Surveillance Annual Report (2020).

[6] Hao X., Chen Q., Pan H., Qiu J., Zhang Y., Yu Q., Han Z., Du X. (2023). Enhancing Drug–Drug Interaction Prediction by Three-Way Decision and Knowledge Graph Embedding. Granul. Comput..

[7] Chen L., Zhang J., Sun Y., Zhao Y., Liu X., Fang Z., Feng L., He B., Zou Q., Tracey G.J. (2023). A Phase I Open-Label Clinical Trial to Study Drug-Drug Interactions of Dorzagliatin and Sitagliptin in Patients with Type 2 Diabetes and Obesity. Nat. Commun..

[8] Chung H., Yu K.-S., Hong K.T., Choi J.Y., Hong C.R., Kang H.J., Park K.D., Shin H.Y., Lee S. (2017). A Significant Influence of Metronidazole on Busulfan Pharmacokinetics: A Case Report of Therapeutic Drug Monitoring. Ther. Drug Monit..

[9] Saeidnia S., Manayi A., Abdollahi M. (2015). From in Vitro Experiments to in Vivo and Clinical Studies; Pros and Cons. Curr. Drug Discov. Technol..

[10] Yang Z., Zhong W., Lv Q., Yu-Chian Chen C. (2022). Learning Size-Adaptive Molecular Substructures for Explainable Drug–Drug Interaction Prediction by Substructure-Aware Graph Neural Network. Chem. Sci..

[11] Hotho A., Nürnberger A., Paaß G. (2005). A Brief Survey of Text Mining. J. Lang. Technol. Comput. Linguist..

[12] Han K., Cao P., Wang Y., Xie F., Ma J., Yu M., Wang J., Xu Y., Zhang Y., Wan J. (2022). A Review of Approaches for Predicting Drug–Drug Interactions Based on Machine Learning. Front. Pharmacol..

[13] Tari L., Anwar S., Liang S., Cai J., Baral C. (2010). Discovering Drug–Drug Interactions: A Text-Mining and Reasoning Approach Based on Properties of Drug Metabolism. Bioinformatics.

[14] Gómez-Bombarelli R., Wei J.N., Duvenaud D., Hernández-Lobato J.M., Sánchez-Lengeling B., Sheberla D., Aguilera-Iparraguirre J., Hirzel T.D., Adams R.P., Aspuru-Guzik A. (2018). Automatic Chemical Design Using a Data-Driven Continuous Representation of Molecules. ACS Cent. Sci..

[15] He H., Chen G., Yu-Chian Chen C. (2022). 3DGT-DDI: 3D Graph and Text Based Neural Network for Drug-Drug Interaction Prediction. Brief. Bioinform..

[16] Vilar S., Uriarte E., Santana L., Lorberbaum T., Hripcsak G., Friedman C., Tatonetti N.P. (2014). Similarity-Based Modeling in Large-Scale Prediction of Drug-Drug Interactions. Nat. Protoc..

[17] Ryu J.Y., Kim H.U., Lee S.Y. (2018). Deep Learning Improves Prediction of Drug–Drug and Drug–Food Interactions. Proc. Natl. Acad. Sci..

[18] Jonsdottir S.O., Jorgensen F.S., Brunak S. (2005). Prediction Methods and Databases within Chemoinformatics: Emphasis on Drugs and Drug Candidates. Bioinformatics.

[19] Schütt K., Kindermans P.-J., Sauceda Felix H.E., Chmiela S., Tkatchenko A., Müller K.-R. (2017). SchNet: A Continuous-Filter Convolutional Neural Network for Modeling Quantum Interactions. Advances in Neural Information Processing Systems.

[20] Li P., Wang J., Qiao Y., Chen H., Yu Y., Yao X., Gao P., Xie G., Song S. (2021). An Effective Self-Supervised Framework for Learning Expressive Molecular Global Representations to Drug Discovery. Brief. Bioinform..

[21] Jaeger S., Fulle S., Turk S. (2018). Mol2vec: Unsupervised Machine Learning Approach with Chemical Intuition. J. Chem. Inf. Model..

[22] Mikolov T., Chen K., Corrado G., Dean J. (2013). Efficient Estimation of Word Representations in Vector Space. arXiv.

[23] Ross J., Belgodere B., Chenthamarakshan V., Padhi I., Mroueh Y., Das P. (2022). Large-Scale Chemical Language Representations Capture Molecular Structure and Properties. Nat. Mach. Intell..

[24] Zhang Z., Bian Y., Xie A., Han P., Zhou S. (2024). Can Pretrained Models Really Learn Better Molecular Representations for AI-Aided Drug Discovery?. J. Chem. Inf. Model..

[25] Haroon S., Hafsath C.A., Jereesh A.S. (2023). Generative Pre-Trained Transformer (GPT) Based Model with Relative Attention for de Novo Drug Design. Comput. Biol. Chem..

[26] Chithrananda S., Grand G., Ramsundar B. (2020). ChemBERTa: Large-Scale Self-Supervised Pretraining for Molecular Property Prediction. arXiv.

[27] Graves A., Schmidhuber J. (2005). Framewise Phoneme Classification with Bidirectional LSTM and Other Neural Network Architectures. Neural Netw..

[28] Taud H., Mas J.F., Camacho Olmedo M.T., Paegelow M., Mas J.-F., Escobar F. (2018). Multilayer Perceptron (MLP). Geomatic Approaches for Modeling Land Change Scenarios.

[29] Huang K., Xiao C., Hoang T.N., Glass L., Sun J. CASTER: Predicting Drug Interactions with Chemical Substructure Representation. Proceedings of the AAAI Conference on Artificial Intelligence.

[30] Marinka Z., Sosi R., Maheshwari S., Leskovec J. (2018). BioSNAP Datasets: Stanford Biomedical Network Dataset Collection.

[31] Wishart D.S., Knox C., Guo A.C., Cheng D., Shrivastava S., Tzur D., Gautam B., Hassanali M. (2008). DrugBank: A Knowledgebase for Drugs, Drug Actions and Drug Targets. Nucleic Acids Res..

[32] Menard S. (2002). Applied Logistic Regression Analysis.

[33] U.S. Food and Drug Administration (2024). XOLREMDI (Mavorixafor).

[34] U.S. Food and Drug Administration (2024). PIVYA (Pivmecillinam).

[35] U.S. Food and Drug Administration (2024). REZDIFFRA (Resmetirom).

[36] Bajusz D., Rácz A., Héberger K. (2015). Why Is Tanimoto Index an Appropriate Choice for Fingerprint-Based Similarity Calculations?. J. Cheminform..

[37] Riniker S., Landrum G.A. (2013). Similarity Maps—A Visualization Strategy for Molecular Fingerprints and Machine-Learning Methods. J. Cheminformatics.

[38] Kheshti R., Aalipour M., Namazi S. (2016). A Comparison of Five Common Drug–Drug Interaction Software Programs Regarding Accuracy and Comprehensiveness. J. Res. Pharm. Pract..

[39] Xiong G., Yang Z., Yi J., Wang N., Wang L., Zhu H., Wu C., Lu A., Chen X., Liu S. (2022). DDInter: An Online Drug–Drug Interaction Database towards Improving Clinical Decision-Making and Patient Safety. Nucleic Acids Res..

[40] (2024). RDKit Documentation. https://www.rdkit.org/docs/.

[41] Bienfait B., Ertl P. (2013). JSME: A Free Molecule Editor in JavaScript. J. Cheminformatics.

[42] Narayan S. (1997). The Generalized Sigmoid Activation Function: Competitive Supervised Learning. Inf. Sci..

[43] Takase S., Kiyono S. (2023). Lessons on Parameter Sharing across Layers in Transformers. arXiv.

[44] Liu Y., Ott M., Goyal N., Du J., Joshi M., Chen D., Levy O., Lewis M., Zettlemoyer L., Stoyanov V. (2019). RoBERTa: A Robustly Optimized BERT Pretraining Approach. arXiv.

[45] Irwin J.J., Shoichet B.K. (2005). ZINC—A Free Database of Commercially Available Compounds for Virtual Screening. J. Chem. Inf. Model..

[46] Kim S., Chen J., Cheng T., Gindulyte A., He J., He S., Li Q., Shoemaker B.A., Thiessen P.A., Yu B. (2020). PubChem in 2021: New Data Content and Improved Web Interfaces. Nucleic Acids Res..

[47] Torrey L., Shavlik J. (2010). Transfer Learning. Handbook of Research on MachiIne Learning Applications and Trends: Algorithms, Methods, and Techniques.

[48] Ahmad W., Simon E., Chithrananda S., Grand G., Ramsundar B. (2022). ChemBERTa-2: Towards Chemical Foundation Models. arXiv.

[49] Gage P. (1994). A New Algorithm for Data Compression. C. Users J..

[50] Hochreiter S., Schmidhuber J. (1997). Long Short-Term Memory. Neural Comput..

[51] Graves A. (2012). Long Short-Term Memory. Supervised Sequence Labelling with Recurrent Neural Networks.

[52] Nair V., Hinton G.E. Rectified Linear Units Improve Restricted Boltzmann Machines, In Proceedings of the 27th International Conference on International Conference on Machine Learning, Haifa, Israel, 21–24 June 2010.

[53] Loshchilov H., Hutter F. Decoupled weight decay regularization. Proceedings of the International Conference on Learning Representations.

[54] Kim S., Jin D., Lee H. (2013). Predicting Drug-Target Interactions Using Drug-Drug Interactions. PLoS ONE.

[55] Vilar S., Harpaz R., Uriarte E., Santana L., Rabadan R., Friedman C. (2012). Drug—Drug Interaction through Molecular Structure Similarity Analysis. J. Am. Med. Inform. Assoc..

