# Supplementary material for: PTB-DDI: An Accurate and Simple Framework for Drug–Drug Interaction Prediction Based on Pre-Trained Tokenizer and BiLSTM Model
Source: Int J Mol Sci. 2024 Oct 23;25(21):11385. doi: 10.3390/ijms252111385 (PMC11546514; doi:10.3390/ijms252111385)
Supplement: Supplementary file 1 [file ijms-25-11385-s001.zip › ijms-3243505-supplementary.pdf]

Table S1 shows the primary hyperparameter setting of the PTB-DDI framework. The max sequence length of token IDs (TL) generated by the ChemBerta tokenizer is 512. The AdamW [45] optimizer with a learning rate (LR) of 2e-5 and weight decay (WD) of 2e-4 on the BIOSNAP dataset, while it has a learning rate of 2e-5 and weight decay of 1e-2 on DrugBank. The batch size (BS) is eight for the BIOSNAP and 16 for the larger DrugBank dataset. The step learning rate (StepLR) scheduler has a gamma (G) of 0.8 and a step size (S) of 10. The total number of training epochs (E) is 30. In the BiLSTM model, the input layer has 512 units, and the hidden layer dimension is 256. In the MLP module, the input layer has 256 neurons, the hidden units are 128, and the output layer has four units. In the predictor, the input dimension of the linear layer is eight and the output dimension is 1.

Table S1. Hyperparameter setting of the PTB-DDI framework on datasets

Hyperparameters / Dataset	TL	LR	WD	G	BS	S	E
BIOSNAP	512	2e-5	2e-4	0.8	8	10	30
DrugBank	512	2e-5	1e-2	0.8	16	10	30

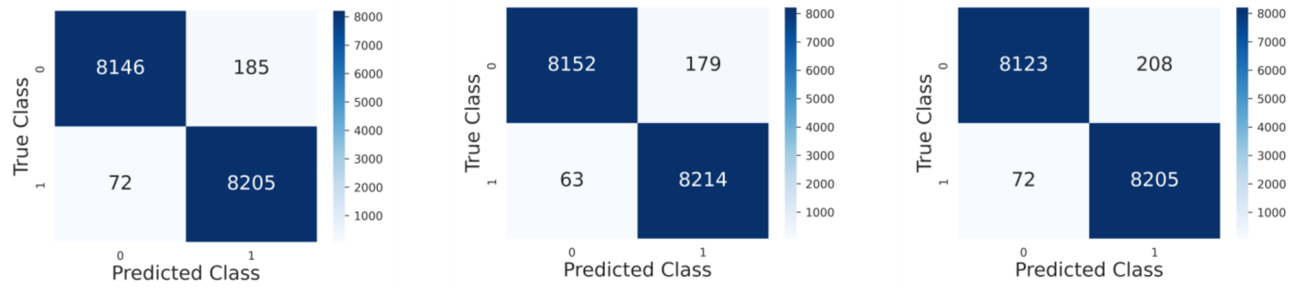

(a) Parameter-sharing

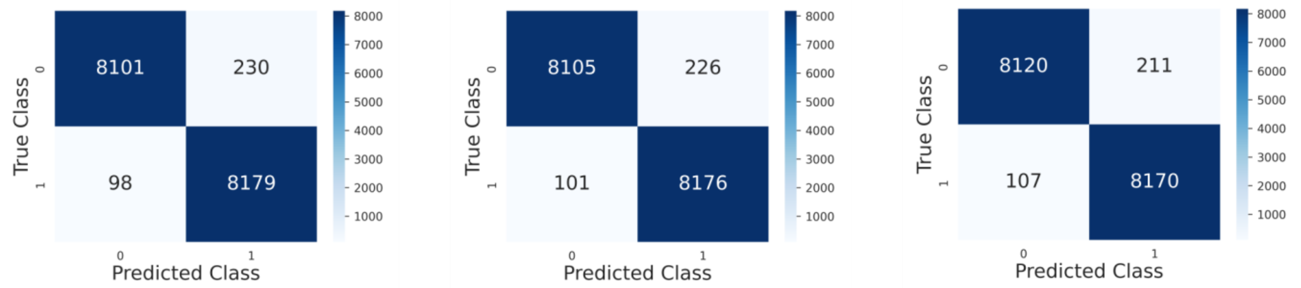

(b) Parameter-independent

Figure S1. It depicts the three parallel experimental results of the PTB-DDI framework regarding the confusion matrix on the BIOSNAP datasets. There are (a) parameter-sharing and (b) parameter-independent prediction results where the x-axis is the predicted value, and the y-axis represents the true label.

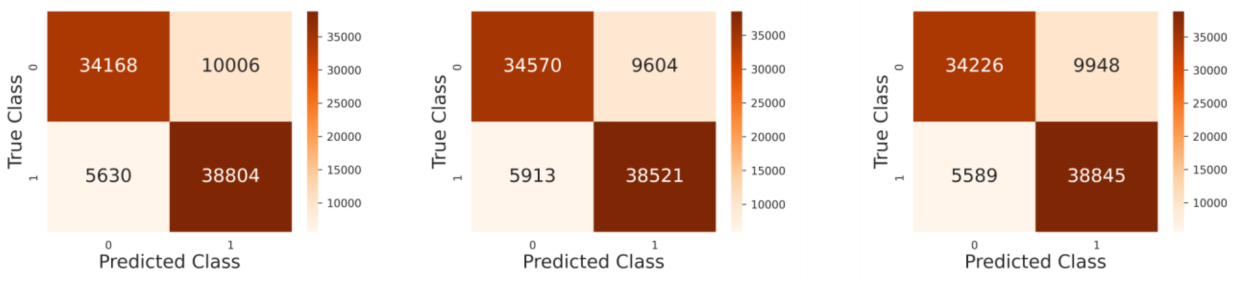

(a) Parameter-sharing

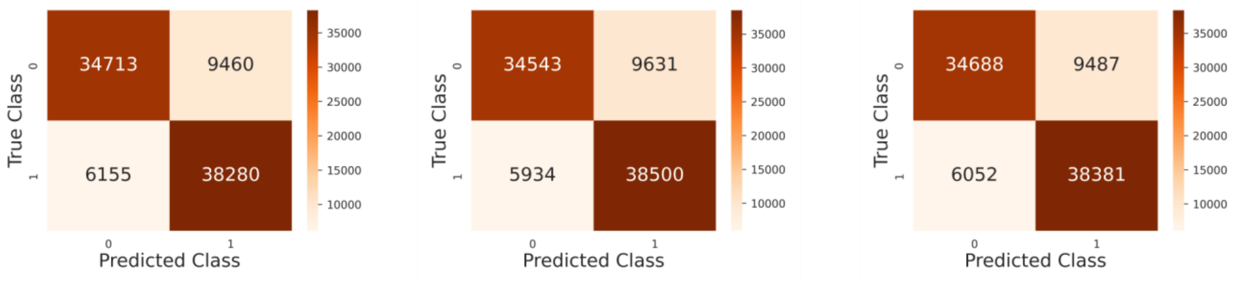

(b) Parameter-independent

Figure S2. It depicts the three parallel experimental results of the PTB-DDI framework regarding the confusion matrix on the DrugBank datasets. There are (a) parameter-sharing and (b) parameter-independent prediction results where the x-axis is the predicted value, and the y-axis represents the true label.
